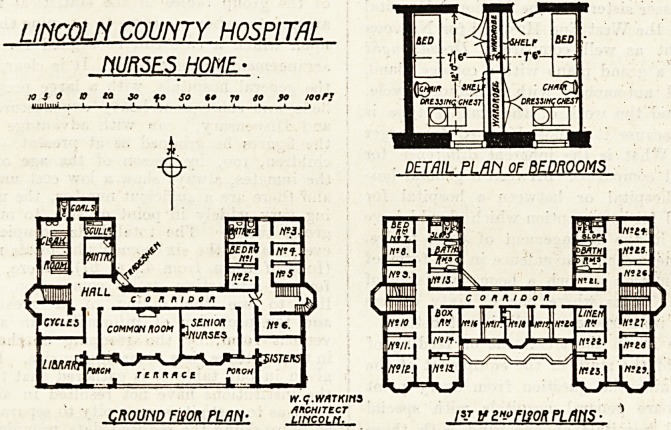# Lincoln County Hospital

**Published:** 1916-03-04

**Authors:** 


### HOSPITAL ARCHITECTURE AND CONSTRUCTION.

### Lincoln County Hospital.

### NURSES' HOME.

This building is detached and stands about 150 yards north of the main hospital block. It is planned in the form of an H, with the sides running north and south. All the sitting rooms face due south and the bedrooms face either east or west, so that every room gets sunshine at some time of the day.

On the ground floor is a large common room for nurses, with a smaller one for junior nurses; both these open on to a wide terrace, and are provided with bay windows. At one end of the south front is a library, at the other a sitting room for sisters. Six bedrooms, with bath and w.c., are provided on this floor in the east wing. In the west wing is a cycle room; cloak room fitted with numbered cloak hooks, each with boot and shoe locker underneath; pantry fitted with small range for occasional and emergency use, a small scullery for dealing with afternoon teas, and a coal store. On each of the two upper floors are twenty-three bedrooms, four bathrooms, four w.c.s and two housemaids' closets, also a linen room and a box-room. The two larger rooms, with bay windows, are allotted to sisters. Six only of the bedrooms are provided with fireplaces, the remainder having hot-water pipes running through them.

The bedrooms are provided with fixed wardrobes ingeniously dovetailed in to save space; the clear width of the room, 7 feet 6 inches, seems rather inadequate, and would appear more so if the bed were drawn on the plan the usual width of 3 feet, instead of about 2 feet 6 inches.

The home, together with a new ward added to the hospital, was erected as a memorial to King Edward VII.

The architect is Mr. W. G. Watkins, of Lincoln.

## Figures and Tables

**Figure f1:**